# Supplementary material for: Are carbon emissions trading and green financial instruments synergistic? -Comprehensive quantitative research based on content analysis
Source: PLoS One. 2024 Mar 7;19(3):e0298601. doi: 10.1371/journal.pone.0298601 (PMC10919601; doi:10.1371/journal.pone.0298601)
Supplement: S1 Data — (ZIP) [file pone.0298601.s001.zip › Data/地级市Z_F工作报告.zip!/╜¡╬≈/╥╦┤║╩╨/└┤╫╘╣1⁄2╓┌║┼í╛┬φ┐╦╩2╛▌═°í┐_2012.docx]

各位代表：

现在，我代表市人民政府向大会报告工作，请予审议，并请市政协委员和列席会议的同志提出意见。

一、2011年工作回顾

2011年，全市人民在省委、省政府和市委的正确领导下，深入落实科学发展观，始终坚持赶超发展不动摇，全面开启幸福宜春建设新征程，凝心聚力，开拓进取，扎实工作，完成了市二届人大六次会议确定的主要任务，实现了“十二五”良好开局。

--致力增总量。全市完成生产总值1078亿元，增长12.8%；财政总收入149.1亿元，增长38.2%，其中地方财政收入92.3亿元，增长39.3%，两项增幅均列全省第3；税收占财政总收入的比重为88.7%，高出全省平均水平5.5个百分点，列全省第3；500万元以上固定资产投资779.5亿元，增长30%；规模以上工业增加值386.5亿元，增长21.7%。县域经济实力增强，其中丰城财政总收入超30亿元，居“全国百强县”第97位、比上年前移2位，并首次挺进“中部十强县”；樟树超20亿元，继续跻身“全省十强”；高安超15亿元；上高、万载均超10亿元。尤其是，经济发展实现“一大赶超、五大突破”：工业经济强势赶超，规模以上工业增加值增幅列全省第1、比上年前移2位，总量列全省第4，超额完成省下达的目标任务；生产总值突破千亿大关；财政总收入突破10亿元县市区达6个，比上年增加2个；外贸出口额突破10亿美元；奉新金源纺织和庆丰包装分别在香港、加拿大成功上市，实现我市企业在境外、国外上市的历史性突破；年接待游客总人数突破1000万人次，旅游总收入达到86.5亿元。

--着力攻项目。工业“三年强攻战”全面推进。深入开展产业升级年和产业招商百日活动，建立健全重大项目推进机制，抓项目、兴工业、强产业成为全市上下的共识共为，全市重大项目总数达217个，其中10亿元以上工业项目71个。锂电新能源产业累计签约项目125个，其中10亿元以上项目23个，实际进资47.3亿元，已进资、动工、投产企业75家。兴发铝业、联威新能源、福斯特新能源、宏宇能源、四特“两城”、泰明光伏等一批重大项目先后竣工投产。樟树荣获“全国金属家具产业基地”称号；丰城获批全省县市区首个省级高新技术产业园区；高安陶瓷产业加快转型升级，跻身全国五大陶瓷主产区之一。江特电机、济民可信、仁和集团等一批骨干企业快速发展。城建“三年大会战”纵深推进。全市在建项目240个，竣工项目144个，累计完成投资328亿元，其中，中心城区在建项目69个，竣工项目48个，累计完成投资177亿元。全市城镇化率达42.7%，提高1.6个百分点。中心城区“人口五年倍增”计划顺利完成，建成区面积达55平方公里，人口超过50万，以第一名的成绩荣获“江西省生态园林城市”称号，并蝉联“中国休闲城市”和“最佳休闲养生城市”。坚持“用管理财政理念管理土地”，土地管理“十坚持、十不批”和国土资源“多快好省”工作机制被国土资源部列为创新典型。农业产业化升级战有效推进。五大重点产业产值366.1亿元，增长27%，农业招商总投资额241亿元，落户项目171个。市级以上农业产业化龙头企业达到215家，上高金农米业、樟树天齐堂中药饮片公司获批国家级龙头企业；农民专业合作社达到1642家，其中省级示范社65家。丰城获批国家农业产业化示范基地，填补了我市无国家级农业产业化示范基地的空白。全市粮食总产39.4亿公斤，粮食收购总量14.9亿公斤，生猪出栏671万头，农机总动力700万千瓦，均居全省首位，丰城、高安荣获“全国粮食生产先进县”，樟树、奉新、上高荣获“全省粮食生产先进县”，分别受到国务院和省政府的表彰。全面推进农田水利基本建设，开工建设水利项目485项，总投资近20亿元，其中，中央、省级投资14亿元。铜鼓列入国家农业综合开发县，实现我市农业综合开发项目县“全覆盖”。奉新在全省农村率先告别“低电压”。

--努力活机制。国有工业企业改革全面完成，国有非工、集体企业改革目标基本实现。扎实推进医药卫生体制改革，城乡居民基本医疗保险参保人数突破500万，乡镇卫生院和社区卫生服务中心全面实施基本药物制度。财税、国有林场、农垦企业、事业单位分类改革稳步推进。科技创新能力增强，组织申报国家、省级科技项目100余项，宜春远大化工“聚氨酯类产品的生物-化学组合合成技术”列入国家“863”计划项目，全市高新技术产业增加值达到72亿元。深入开展“质量兴市”活动，省级锂电产品检测中心获批筹建，樟树新增2个全国驰名商标，商标品牌总量居全省县市区第一。开放型经济成效显著，实际利用外资4.3亿美元，增长19.3%；引进省外5000万元以上项目137个，项目数连续四年居全省之首。金融机构新增贷款接近百亿元，银行业存、贷款余额和总资产均实现或接近三年翻番。成功举办第五届月亮文化节、第六届中国竹文化节、奉新百丈清规文化节等大型活动，宜丰、奉新被授予“中国竹产业集群”称号。

--全力保民生。全市民生支出达到150亿元，占财政总支出的70.5%。城镇居民人均可支配收入16431元，增长14.6%；农民人均纯收入6981元，增长20.4%。全面完成省政府下达的各项民生工程任务，城乡低保、城镇新增就业、创业型城市创建、小额贷款、残疾人社区康复等工作走在全国、全省前列。保障性住房建设在全省率先全面开工，当年开工9025套、竣工9284套，开发用地配建保障房“四定一限”的供地模式向全省推广。明月山机场、靖安洪屏抽水蓄能电站等重大基础工程加快推进，杭长铁路客运专线、宜春汽车客运总站等项目建设进展顺利，昌铜高速昌奉段竣工通车，农村公路综合服务站建设在全省率先集中开工，国省道干线公路养护管理“迎国检”工作获全省第一。建立“菜篮子”市场价格补偿机制，确保了市场价格平稳。社会事业加快发展，全市新、扩建中小学校、幼儿园14所，增加学生容量2万余人；普通高考二本上线人数再创历史新高，靖安荣获“全省教育工作综合督导先进单位”，铜鼓荣获“全省义务教育均衡发展示范县”，中心城区通过省政府国家二类城市语言文字工作验收；宜春学院获批药学硕士专业学位授予权；宜春电大荣获“全国广播电视大学优秀教务集体”。医疗服务能力有效提升，率先在全省实现新农合支付方式全覆盖，参合农民突破400万人，参合率达98.3%，宜春市人民医院荣获“全国文明单位”。“七城会”羽毛球和女子排球赛事圆满承办。中心城区“文化之窗”建成并即将投入使用，新建乡镇综合文化站162个、农家书屋750家。第八届村（居）委会换届工作平稳有序。环境质量持续改善，靖安九岭山自然保护区晋升为国家级自然保护区。开展了以“三同四民”为主题的万名干部下基层活动，广大群众交口称赞。深入推进“全民献计出力、共建幸福宜春”活动，全民共建幸福宜春的氛围日趋浓厚。加快城区电子监控体系建设，治安防控能力进一步增强，公众安全感测评继续居全省前列。加大交通安全等专项整治力度，安全生产保持稳定向好态势。

--合力树形象。深入开展“发展提升年”活动，连续两年获全省考评第1，奉新被评为“全国中小企业创新服务先进单位”。“五五”普法获全国先进， “六五”普法全面启动。进一步规范和完善市政府会议、公文审批等工作制度，促进了政府工作高效、规范、有序运转。坚持向人大报告政府工作，认真接受人大法律监督和政协民主监督，82件人大代表建议、168件政协委员提案全部办复。加快电子政务建设，完善政府信息公开，创办《在线访谈》的经验做法在全省推广。全面推进网上审批和电子监察系统建设，拓宽重大项目审批绿色通道，工作效能和服务水平不断提升。进一步强化监察审计工作，党风廉政建设继续加强。国防后备力量和民兵预备役建设、人民防空、国家安全、外事侨务、民族宗教、涉台事务、邮政通信、石油、烟草、盐业、气象水文、防震减灾、无线电管理、妇女儿童、统计、档案、扶贫、老龄、残疾人和红十字会等工作都取得了新成绩。

各位代表，一分耕耘一分收获，我们深深懂得，奋斗伴随艰辛，成绩来之不易。过去的一年，我们上下一心，团结拼搏，用辛勤和汗水谱写了赶超发展的新篇章，迈出了幸福宜春建设的新步伐。这些成绩的取得，得益于省委、省政府和市委的坚强领导，得益于历届班子打下的坚实基础，得益于全市上下的奋力拼搏。在此，我代表市人民政府，向付出辛勤劳动的全市人民，向给予政府工作大力支持的人大代表和政协委员，向各民主党派、工商联、无党派、人民团体、驻宜部队、公安民警、武警官兵和社会各界人士，向所有关心支持宜春经济社会发展的同志们、朋友们，表示崇高的敬意和衷心的感谢！

在看到成绩的同时，我们也清醒地认识到发展中的困难和问题：经济总量不大，发展不快、发展不够仍是最主要的矛盾；中心城区和县城辐射带动力不强，基础配套功能有待完善；社会不稳定因素较多，安全维稳压力大；政府自身建设还需进一步加强，等等。对此，我们将高度重视，采取切实有效措施，认真加以解决。

二、2012年目标要求

今年是加速宜春进位赶超的关键之年，也是新一届政府全面履职的起始之年。五年看头年，初战务必胜。面对新的形势、新的挑战，我们要冷静分析，积极应对。一方面，国家继续实施积极的财政政策和稳健的货币政策，财政重点投向新兴产业、农田水利、保障房建设等领域；今后10年，全省将投入2200亿元用于兴修水利；省委、省政府召开了鄱阳湖生态经济区建设推进大会，发出了“五年见成效，十年大跨越”的总号令，为我们争取更多的项目和资金提供了难得的政策机遇。另一方面，杭长铁路客运专线建设加快推进，明月山机场即将竣工通航，“中部之心”的交通优势更加凸显，将为加快开放型经济发展插上腾飞之翼。同时，我们也要看到，全省上下竞相发展、相互赶超的态势更加激烈，稍有松懈，不但难以进位，甚至有掉队的危险。竞争不同情落后，机遇不垂青弱者，“狭路相逢勇者胜”。我们惟有牢牢把握“稳中快进、难中取胜”的基调，突出发展主题不动摇，锁定发展目标不放松，善于在应对挑战中把握机遇，在改革创新中破解难题，在多重压力下拼力加速，奋力推动全市经济更大跨越、更快赶超，才能不辜负人民的重托，才能无愧于时代赋予的光荣使命！

根据中央、全省的部署和要求，结合宜春实际，今年政府工作的总体要求是：全面贯彻党的十七大、十七届六中全会和中央、全省经济工作会议精神，以邓小平理论和“三个代表”重要思想为指导，深入贯彻落实科学发展观，围绕“建设幸福宜春”的总目标和“保增长、兴产业、惠民生、促和谐”的总要求，按照打造“亚洲锂都、宜居城市、森林宜春、月亮之都”的发展定位，准确把握和紧密跟踪国家宏观调控新走向，以更大的力度推进兴工强市、产业升级，以更大的力度推进城市建设和城镇化进程，以更大的力度推进农业产业化、现代化，以更大的力度改善和保障民生，确保经济主要指标赶超进位，确保社会和谐稳定，以优异成绩迎接党的十八大胜利召开。

全市经济社会发展主要预期目标是：生产总值增长12%，达到1250亿元；财政总收入增长18%，达到176亿元，其中地方财政收入增长18%，达到109亿元；规模以上工业增加值增长20%，达到470亿元；全社会固定资产投资增长25%，达到1060亿元；社会消费品零售总额增长16.5%；引进省外5000万元以上工业项目资金增长15 %；实际利用外资增长10%；外贸出口增长5%；城乡居民收入增长12%；城镇化率提高2个百分点；城镇登记失业率控制在4.5 %以内，人口自然增长率控制在8‰以内。

各位代表，上述目标是一个预期目标，在实际工作中，我们将自我加压，排难奋进，按照进位赶超的要求和科学理性的原则，促使全市上下以更加积极的姿态，确定更高的工作目标，争取更好的发展成果！

三、2012年工作任务

建设幸福宜春，必须始终围绕做大经济总量、尽快提高占全省的比重这一总体要求，深入打好工业“三年强攻战”、城镇建设“三年大会战”、农业产业化升级战和县域经济发展“三年大竞赛”，全面实施 “十大幸福工程”，为幸福宜春建设开好局、起好步。

--紧抓重大项目不放松。坚定不移地实施“兴工强市”战略，以“决战工业园，跨越两千亿”为核心，深入开展“主攻重大项目、推进产业升级”活动，突出“四抓”：抓重大项目线索。理出一批重大项目线索，制定专门方案，集中力量，加强调度，高效服务。设立重大项目线索奖和市政府重大项目华木莲奖，分别对引进10亿元以上的重大项目提供线索的有功人员进行奖励；对重大项目引进，促其落地、建设、投产并达产达标的有功单位和个人予以重奖。抓重大项目引进。围绕主导产业、资源优势、产业政策，建立和完善全市重大产业项目储备库，确保项目动态保留量不少于200个。全市重点发展锂电新能源、医药食品、机械电子三大产业，各县市区结合实际，选择产业重点发展。着力引进一批投资规模大、科技含量高、创税能力强的支柱型重大项目，每个主导产业力争引进1-2个龙头型项目，其中锂电新能源产业力争引进1-2个百亿元以上项目，落地项目100个以上，主营业务收入突破100亿元。跟踪推进深圳比亚迪、宝安集团、康佳集团、香港大宇光电、浙江万华、上高电厂等重大项目。抓重大项目建设。市级重点调度10亿元以上项目，重点抓好100个项目的推进。着力推进市本级青山能源、成品油（库）管道、樟树宏宇能源、靖安洪屏抽水蓄能电站、丰城新高焦化等一批重大项目建设，全年力争100个工业项目开工率达到70%，投产率达到40%。从全市优选出200户发展前景广、成长性好的重点企业，市、县两级各调度100户，做到优先审批、优先用地、优先争资、优先融资、优先招工，促其做大做强。力争宜工集团、四特酒公司、仁和集团主营业务收入均超50亿元，济民可信、丰电、金源纺织、丰矿等龙头企业均超30亿元，超20亿元企业达到7户，超10亿元企业达到15户，年纳税1000万元以上企业达到200户。抓重大项目服务。坚持“今天工业园区、明天城市新区”理念，大力推进基础设施配套建设，加快园区工业废水集中收集治理，加大生态工业园区创建，力争全市工业园区均进入省级生态园区行列，创新中国成立家产业示范基地1-2个；主营业务收入超200亿元园区达到6个，其中，争取丰城工业园区突破500亿元，宜春经济开发区、高安工业园区突破300亿元。加大信贷支持，对重点项目资金实行定期调度，力争成长型企业贷款需求满足率达70%以上，重大工业项目达到100%。坚持集约发展理念，着力提高土地投资强度和产出效益，力争新入园项目土地利用率达到80%以上，亩均投资150万元以上，亩均税收5万元以上，宜春经济开发区和丰城高新技术产业园区创新中国成立家级开发区取得实质性进展。

--推进城市建设不减速。围绕“打造宜居品牌，建设幸福城市”目标，坚持规划先行，强化 “三高”理念，着力在“四大一重”上下功夫：扩大城市控规。突出抓好城区修建性详规和专项规划编制，统筹宜阳新区、宜春经济开发区、明月山温泉风景名胜区发展，努力打造宜居的福地、宜业的热土和宜游的乐园。积极编制丰樟高城市群战略规划，推进丰樟高中等城市群建设；加快其他县新城区建设，理性改造老城区。全面推进小城镇建设，培育发展一批工业强镇、商贸大镇、旅游名镇。拉大城市框架。对中心城路网框架统一规划、统筹建设。以城镇建设三年大会战为载体，抓好全市240个在建重点工程进度，中心城重点加快推进禅博园、“科技之窗”等69个在建工程项目。留足重大项目用地。中心城区重点抓好湖田片区土地收储，推进湖田片区整体开发。切实保护好耕地，实现耕地总量动态平衡。全力打好土地开发大会战，积极推进低丘缓坡、荒滩等未利用土地综合开发利用试点，开展闲置工业用地专项清理，全年争取省重大项目用地指标2万亩、清理闲置土地1.5万亩、盘活土地1万亩、新增用地指标中的50%原则上用于工业发展、新增工业用地5万亩。培育大品牌服务业。着力引进一批规模大、档次高、辐射力强的商贸、物流园区和综合性娱乐设施，带动服务业上档升级。大力发展商贸金融、物流中介等生产性服务项目。抓好粮油、农资、服装、钢材、再生资源等大市场项目规划和建设，支持邮政、通信、石油、烟草、盐业等企业做大做强。围绕打造“温泉之乡、生态家园、禅宗圣地、月亮之都”的旅游总体形象，按照“两区一线一重点”的发展格局，深入实施旅游产业升级战，精心筹办好省第三届花博会和第六届月亮文化节。加快温汤集镇整体规划改造，以天沐温泉度假村为龙头，进一步做大温泉产业。推进明月山创新中国成立家5A级景区和国家级度假区，启动奉新百丈山国家4A级景区创建。力争全市实现年接待游客1250万人次，旅游总收入突破100亿元。加强银行业服务体系建设，争取宜春农村商业银行上半年挂牌营业，全年引进股份制银行2家，金融机构新增贷款突破100亿元。加快推进企业上市，争取2家企业完成股改、1家企业报证监会审批上市。注重投资少、见效快的民生工程项目建设。启动中心城区市容环境和市政基础设施三年大整治与大提升活动，以创新中国成立家生态园林城市为载体，有重点、分步骤对小街小巷、交通拥堵、公共设施、环卫绿化、亮化立面、建筑工地管理等六个方面进行整治提升。

--增创农业优势不懈怠。巩固基础，发挥优势，彰显特色，全力推进“四大战役”：粮食大丰收持久战。认真落实粮食直补、农资综合补贴、良种补贴和农机具购置补贴等政策，进一步激发各级重农抓粮、农民务农种粮、农技人员科技兴粮的积极性，争取粮食总产达到40亿公斤以上。农业产业化升级战。积极开展农业产业招商，确保农业招商引资额占全市招商引资总额的20%以上，每个县市区引进1-3个亿元以上农业招商项目。发展壮大十大优势特色产业，突出抓好粮食、畜禽、高产油茶、毛竹、有机（富硒）农产品五大重点产业，力争五大重点产业产值突破400亿元，市级以上农业产业化龙头企业达到230家，农民专业合作社达到1800家。积极做好现代农业示范区申报，力争成功创新中国成立家级现代农业示范区1个、省级现代农业示范区2个。农田水利设施建设攻坚战。在完成病险水库除险加固任务的同时，突出抓好小港联圩、赣西肖江堤、药湖联圩等重点堤防加固；抓紧潦河、锦北、袁北、丰东等4座大型灌区续建配套与节水改造。加快小农水重点县和高标准农田建设，大力推进农村土地整治项目实施，确保年内新建高标准农田30万亩以上。绿色生态优化战。深入开展全市7条主要河流水环境污染综合治理，争取靖安成功创新中国成立家级生态县。继续实施造林绿化“一大四小”工程，突出抓好昌铜高速通道绿化。策应全省打造赣西千里花卉苗木产业走廊，加快苗林一体化建设，重点抓好奉新、上高、袁州万亩观光示范园建设，大力发展花卉苗木产业。深入推进新农村建设，突出抓好104个和谐小康家园示范村建设。结合城镇化建设，推进新农村建设村镇联动、村落连片，大力实施农村清洁工程，进一步改善村容村貌。

--深化改革创新不犹豫。深入推进各项改革，加快科技创新步伐。在重点改革上主动跟进。继续抓好国有企业改革的扫尾工作，全面推进新一轮企业改革。深化医药卫生体制改革，落实村医补助和保障政策，实现村卫生室基本药物制度全覆盖。深化文化体制改革，加快经营性文化单位转企改制，抓好宜春报业集团、宜春电广传媒的组建。统筹推进事业单位分类改革。加快户籍制度改革，深化集体林权制度配套改革。在发展转变上敢于先行。牢牢把握全球新一轮产业结构调整、全省加快推进鄱阳湖生态经济区建设等机遇，积极争取国家和省级低碳城市建设、资源型城市转型、循环经济和城市矿产建设等试点，拓宽发展空间。在科技创新上力争突破。进一步加大与知名高校、科研院所的战略合作，力争200个产学研科技合作项目落地，各类专利申报数突破1000件。积极筹建奉新纺织产业检测中心和竹加工、玻纤碳素行业工程技术研究中心，大力实施30个高新技术产业化项目，力争全市高新技术产业增加值突破100亿元。深入开展“科技入园、科技进村、科技惠民”活动，完善科技服务体系。推进实施名牌带动战略，新增国家地理标志保护产品、驰名商标各2个，国家级标准化技术委员会1个。在扩大开放上全面搞活。鼓励各类创业主体自主创业，大力支持科技型、创新型和就业型小微企业发展。创新招商引资方式，突出产业链招商，力争全年实际进资突破300亿元。拓宽出口渠道，着力做大总量、提升质量。鼓励和扶持物流产业发展，引进和培育一批大型物流企业，大力发展铁海联运、铁水联运，切实降低企业物流成本。积极争取设立宜春海关，加快宜春锂电、高安建陶、樟树盐化工、万载烟花爆竹等四个口岸作业区建设，增强快速通关能力。推进电子口岸建设，实现通关、物流、外贸、运输监管、口岸服务的信息共享。

--促进民生改善不动摇。坚持以民生为重、民生为先、民生为上，努力使政府开支“瘦身”，民生投入“扩容”，让广大群众更多地享受发展成果。加强就业和社会保障。推进就业再就业工作，鼓励和支持自主创业，突出抓好大中专毕业生和困难群体人员就业。积极做好社会养老保险和医疗保障工作，加大社保、医保扩面征缴力度，确保参保人数和基金征缴稳中有升。推进新型农村养老保险工作并确保实现全覆盖，全面实行高龄老人津贴制度，推动建立养老服务补贴制度，进一步提高城乡敬老院标准化建设水平。加快社会福利事业发展，完善社会救助体系，健全农村留守儿童帮扶机制，加大扶持残疾人康复和就业力度。大力实施重点村整村推进扶贫开发。加快社会事业发展。大力实施全市教育规划纲要“十大工程”建设，制定中心城区教育发展专项规划，积极开展国家级中小学素质教育督导评估试点城市工作，争取成功申报江西省第四届文明城市。进一步改善基层卫生服务条件，规范实施基本公共卫生服务均等化。巩固创卫成果，确保顺利通过国家复审迎检。做好文化资源的挖掘、保护、包装和推介工作，加速文化产业与旅游产业的深度融合式发展，促进文化产业大发展、大繁荣。抓好“文化惠民工程”，加快基层文化设施建设，繁荣文艺创作，丰富群众文化生活。广泛开展全民健身运动，提高竞技体育水平。以优生优育为重点，深化人口计生工作综合改革。继续实施家电下乡惠民工程，力争销售总额突破10亿元。促进社会和谐稳定。落实企业安全生产责任制，加大道路交通、消防等安全专项整治，强化校车安全等各项校园安全措施，提高消防应急救援水平，坚决遏制各类重特大事故发生。加强饮用水源、农产品、食品药品质量安全体系建设，做好重大动物疫病防控。坚持领导干部处理群众来信和接待群众来访制度，突出抓好信访积案化解，切实做好民族宗教工作。加强和创新社会管理，推进城乡社区服务体系建设，深入开展平安创建活动，加快“数字宜春”建设，依法防范和严厉打击各类违法犯罪活动，促进社会治安秩序进一步好转。

在做好上述工作、推进重大项目建设的同时，今年市政府还将着力抓好事关全市经济社会发展、涉及老百姓切身利益的大事、难事和急事，重点抓好20个项目。

（一）全市10个：

1、抓好481座水库除险加固；

2、配合推进杭长铁路客运专线建设；

3、加快昌铜高速奉铜段建设，力争全线通车；

4、开工建设宜万高速公路；

5、新建保障性住房22980套，各类棚户区改造9247套，确保完成省下达的农村危旧房改造任务；

6、新建、改扩建中小学35所，其中中心城区新建、改扩建中小学12所，逐步化解全市城区中小学“大班额”问题；

7、抓好“菜篮子”、“米袋子”工程，全市建设放心粮油、蔬菜供应连锁店20家、生猪活体储备基地1-2个，确保中心城区常年蔬菜基地面积达到1.3万亩；

8、实施农村自来水工程，全年解决30万人的饮水安全问题；

9、新改建硬化农村公路516公里，新增4%的自然村通水泥（油）路；

10、完成520个行政村“广播村村响”工程建设任务，全面实现“村村响”目标。

（二）中心城区10个：

1、争取完成宜春新火车站综合交通枢纽工程主体工程建设；

2、力争明月山机场年底竣工；

3、确保明月大道竣工通车；

4、完成花博园建设；

5、开工建设市人民医院（北院）；

6、加快明月大桥等桥梁建设，打通卢洲北路等断头路，整治改造小街小巷50条，有效解决道路交通拥堵、居民出行难的问题；

7、完善市政基础设施，抓好文体东路等排水设施改造，新改建公厕20座、垃圾中转站8座，居民生活垃圾收集处理率达到95%以上；

8、开展秀江河道综合整治，突出抓好水域清捞保洁、截污排水和清淤疏浚；

9、加强居民小区等部位的视频监控体系建设，实现“天网工程”全覆盖；

10、规划建设集残疾人康复训练、就业培训及托养等一体的市残疾人综合服务中心。

对以上20个项目，我们将按照“项目化、时间表、责任人”的要求，确定责任主体，明确完成时限，抓好督促落实，确保向全市人民交一份满意的答卷。

各位代表，美好的发展前景，给了我们宽广的创业舞台。面对时代的选择、人民的期望，我们倍感责任重、压力大，惟有一丝不苟干工作，一着不让抓发展，一尘不染守气节，不断推动政府管理创新，努力建设人民满意的服务型政府。做到求实、务实、落实。大力弘扬实干精神，坚持少发文、少开会，做到重实践、重实际；坚持抓具体、抓重点、抓实效，做到“花开有果”、“掷地有声”；坚持督查到位、评判到位、奖惩到位，抓住不落实的事，追究不落实的人，让实干者有想头、苦干者有甜头。做到守责、负责、问责。牢固树立“负责如负山、守责如守城、履责如履冰”的意识，务求履责必实、失责必究、问责必严。自觉接受人大法律监督和政协民主监督。加强审计监督、行政监察，健全考评机制。深入开展“集中整治影响发展环境的干部作风突出问题”活动，进一步优化工作流程，创新服务方式，提高办事效率，打造“零缺陷”服务。做到亲和、平和、融和。坚持精诚合作、团结共事，坦诚相处、真诚以待。认真落实廉政建设各项规定，深入推进风险岗位廉能管理，严肃查办各类违法违纪案件，从严控制财政支出，以平和的心态对待名利得失，以饱满的热情投入干事创业，以优良的政风赢得群众的信任和支持。

各位代表，幸福宜春的美好前景令人鼓舞，进位赶超的宏伟目标催人奋进。让我们在市委的坚强领导下，以“锐气藏于胸，才气行于事，正气施于人”的胸襟，谋务实之举，鼓实干之劲，尽为民之责，锐意进取，克难攻坚，为创造宜春更加美好的明天而不懈奋斗！
